# Enhancing the Interaction Between Pd Thin Films and Hydrogen via Atomic Stepped Interface Structures

**DOI:** 10.3390/ma19030596

**Published:** 2026-02-03

**Authors:** Yanxia Liang, Linghui Hou, Xinhua Ma, Dahai Liu, Hui Zhao, Tong Shi, Yong Fan, Wuyun Xiao

**Affiliations:** 1 State Key Laboratory of Chemistry for NBC Hazards Protection, Beijing 102205, China; yxliang0701@126.com (Y.L.); hlh@whu.edu.cn (L.H.); coralhua@126.com (X.M.); l_dahai@126.com (D.L.); nicholas_chao05@163.com (H.Z.); 2 Yantai Research Institute, Harbin Engineering University, Yantai 265503, China; t.shi1@hrbeu.edu.cn

**Keywords:** stepped interface, thin films, magnetron sputtering, hydrogen, columnar crystal

## Abstract

Highly active interfaces are crucial to the hydrogen adsorption performance of nanomaterials. However, it remains challenging to conveniently and efficiently regulate atomic stacking characteristics. Here, we present a straightforward yet effective strategy for generating a high density of stepped atoms at the surface of thin films by controlling the migration behavior of sputtered atoms during deposition. Tuning sputtering power and substrate temperature yields wide-scale stepped interface structures, thus generating irregular conical columnar nanocrystals. Benefiting from the active and stable stepped atoms at the zigzag interface, the samples exhibit an excellent threshold pressure at 200 °C and a hydrogen adsorption of 110.06 cm^3^/g at 6 MPa, which is 2.2 times higher than that of conventional Pd thin films. Based on the control of nucleation and crystal growth during magnetron sputtering deposition, this method provides appropriate energy for surface atomic migration on columnar crystals, achieving high-density stepped interface structures. It can be readily extended to other substrates and noble metal systems, thus offering a novel strategy and guidance for the design of efficient and cost-effective hydrogen-interactive materials.

## 1. Introduction

Adequate and reliable energy supply plays a pivotal role in societal development. Hydrogen energy has been widely recognized as a benchmark for advanced renewable energy [1,2,3,4]. However, the technologies for its production, storage, and safe utilization are not yet fully mature. The absence of commercially and industrially viable electrode and storage materials is the major bottleneck hindering its widespread application [5,6]. Enhancing noble metal utilization efficiency in reaction and storage systems to reduce costs is a critical challenge.

Significant advances have been made in developing nanomaterials for hydrogen applications. Though Pd and Pt-based materials have demonstrated promising catalytic performance with the continuous optimization of structure and activity, they still fail to meet the requirements for widespread deployment [7,8]. Studies have demonstrated that the nanosize effect of powders can effectively enhance reaction performance due to a significant increase in specific surface area [9,10,11]. However, the non-self-supporting nature and inevitable self-aggregation of nanopowders compromise their reliability, hindering practical applications [12]. Nano-thin-film materials possess advantages including high stability, controllable cost, and high single-atom utilization efficiency [13,14]. However, their performance improvement is primarily constrained by the surface morphological structures and the density of active sites.

In recent years, atomic-scale interface engineering has attracted widespread attention for enhancing the intrinsic activity of nanomaterials, owing to its abundant active sites and strong electronic effects [15,16,17]. Nevertheless, the cumbersome synthesis processes and thermodynamic instability factors make it practically difficult to manipulate atomic stacking characteristics at the interface. Therefore, it is essential to explore effective and straightforward strategies for tuning the atomic interfaces of transition metals and to unravel the underlying mechanisms governing interactions between interface structures and hydrogen.

Magnetron sputtering is one of the most well-established techniques for fabricating high-quality thin films and widely applied in the preparation of biomaterials, corrosion-resistant materials and catalytic materials [18,19,20,21]. Driven by collisions with energetic incident atoms and the external electromagnetic field, target atoms are converted into sputtered atoms and gradually deposited onto the substrate surface [19]. Notably, by tuning the density of incident atoms and the deposition energy of sputtered atoms during film formation, we can modulate energy transfer, diffusion, and accumulation, thus controlling the nucleation modes and growth processes of sputtered atoms, as well as regulating the formation of crystalline and amorphous phases [22,23]. This offers the possibility of introducing nanocrystals with controllable morphology, grain size, and volume fraction into transition metal materials. More importantly, through the exploitation of columnar crystal formation ability and unique kinetic deposition effect at different temperatures, we can modulate the nucleation and growth of in-situ formed nanocrystals to retain the desired active interface structures during film formation.

To validate this concept, this study reports a method for fabricating thin films enriched with active interface structures by manipulating the deposition behavior of sputtered atoms, yielding nanocrystals with both micro-nano- and sub-nano-scale wide-range stepped interfaces. The as-prepared thin films with abundant atomic stepped interfaces exhibit excellent activity for hydrogen adsorption, with a maximum capacity of 110.06 cm^3^/g, which is 2.2 times higher than that of conventional Pd thin films. Furthermore, key deposition parameters (such as temperature, sputtering power, and pressure) play a crucial role in modulating the microstructure of films, which in turn affects the thin-film material properties. This study demonstrates that 100 W is the critical sputtering power for fabricating nanocrystalline films with stepped interface structures. The density of stepped interface and columnar grain size are closely related to sputtering power and deposition temperature: low-temperature and high-power deposition conditions enable the fabrication of Pd films with small grain sizes and abundant stepped interface structures. Stepped interface structures and smaller grain sizes not only create a higher density of active reaction sites but also effectively tailor the local electronic structures with low-coordination characteristics, thereby enhancing the hydrogen interaction activity.

## 2. Materials and Methods

### 2.1. Thin Film Deposition and Sample Preparation

Single-layer Pd and Pt films were deposited on Si and Ni substrates by using a high-vacuum magnetron sputtering system (PVD500, SKY Technology Development Co., Ltd., Shenyang, China) with a DC source. The Si substrates were single-crystal, while the Ni substrates were polycrystalline with a grain size of about 1 μm. The Si substrates and Ni substrates were purchased from Fangdao Semiconductor Co., Ltd. (Shenzhen, China) and Zhongnuo Advanced Material Technology Co., Ltd. (Beijing, China), respectively. Thin films were prepared concurrently on Si and Ni substrates under each deposition condition. SEM analysis indicated minimal differences in the microstructure of films on these two substrates. Accordingly, the Pd films on Si substrates are presented as examples. The thickness of the substrates was 1 mm, and the targets used in the system were high-purity (99.99%) elemental Pd. The sputtering targets were purchased from Zhongnuo Advanced Material Technology Co., Ltd. (Beijing, China), with a diameter of 3 inches. The target–substrate distance was set at a constant 15 cm to ensure an appropriate deposition rate and a dense film microstructure while reducing the interference of thermal radiation from the target on the substrate. The base pressure of the chamber was maintained at 4 × 10^−5^ Pa, thus precluding the introduction of extraneous contaminants, oxides, or unintended surface adsorbates during film deposition. During sputtering, the Ar partial pressure was set to 2 Pa and 3 Pa, respectively. The sample stage rotated at 5 rpm to ensure film uniformity. The substrate heating module was integrated into the magnetron sputtering system, which adopted resistance heating. Table 1 presents the Pd thin film samples prepared under different deposition parameters, including sputtering power (samples #1, #2, #3, and #4), substrate temperature (samples #1 vs. #5 and samples #2 vs. #6), and Ar partial pressure (samples #2 vs. #7). The deposition time varied for samples prepared at different sputtering powers to ensure a comparable film thickness. All samples were fabricated in two identical batches under identical experimental parameters, wherein one batch was exclusively designated for structural characterization, and the other was directly employed for hydrogen adsorption performance testing, so as to avoid the interference of characterization procedures on the sample performance.

### 2.2. Thin Film Characterization

The microstructure of the as-prepared films was characterized by a Tecnai F30 (FEI Company, Hillsboro, OR, USA) transmission electron microscope (TEM) with an accelerating voltage of 300 kV. Considering the particularity of the film samples and the focus on surface structure, it is necessary to employ focused ion beam (FIB) techniques for irradiating and cutting the macroscale film samples. The platinum–carbon (Pt-C) layer was deposited on top of the film to protect its surface from damage by the high-energy ion beam. The surface and cross-sectional morphologies as well as the film thickness of the samples were characterized using a scanning electron microscope (SEM), model SEM5000P (Guoyi Quantum Co., Ltd., Hefei, China), with an accelerating voltage of 5 kV. Given the excellent electrical conductivity of Pd itself, no gold sputtering treatment was performed on the film surface for SEM characterization. Statistical analysis of grain sizes was performed with the Nanomeasure software (Version 1.2.0). The phase composition of the films was identified via X-ray diffractometer (XRD, Panalytical Empyrean, Almelo, The Netherlands) using Cu Kα radiation, with a scanning speed of 0.04°/s and an incident angle of 1°. A grazing incidence mode was adopted in the XRD measurement to avoid substrate signal interference. Additionally, adsorption performance tests were conducted on a BSD-PH2001 (BesTech Co., Ltd., Beijing, China) high-pressure gas adsorption-desorption apparatus to evaluate the hydrogen interaction capability of the samples. Prior to formal hydrogen adsorption tests, the samples were degassed at 200 °C under high vacuum in the instrument to remove physisorbed species on the Pd film surface. The pressure range was 0–6.2 MPa, and the temperature was 200 °C. The adsorption curves of each sample were obtained by subtracting the background signal from the film substrate to eliminate the impact of the substrate material.

## 3. Results and Discussion

### 3.1. Stepped Interface Structures and Growth Mechanism of the Thin Films

**Figure 1:**
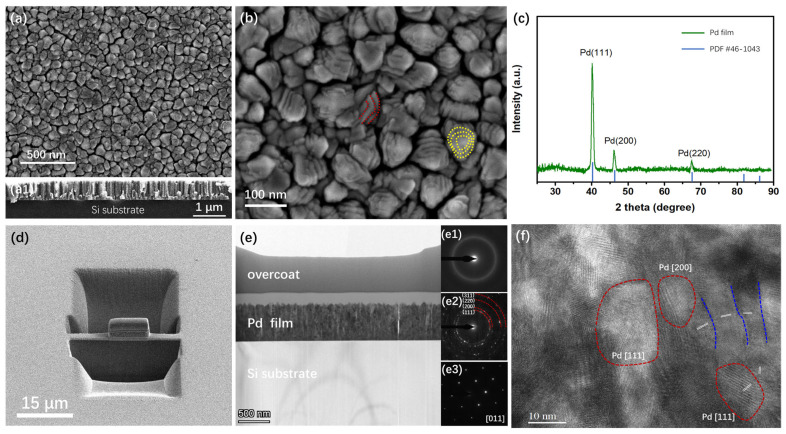
Microstructure of a typical Pd thin film with a stepped surface. SEM image from (**a**) plan-view and (**a1**) cross-sectional view. (**b**) High-magnification SEM image. (**c**) XRD pattern showing the (111), (200), and (220) planes. (**d**) FIB image of the TEM sample. (**e**) TEM image where (**e1**) is the diffraction pattern of the protective layer region, (**e2**) is the diffraction pattern of the film layer region, and (**e3**) is the diffraction pattern of the substrate layer region. (**f**) High-resolution TEM image, in which the regions marked with red dashed lines represent typical grains, the blue dashed lines denote adjacent grain boundaries, and the white solid lines indicate atomic arrangement orientations.

Pd and Pt thin films with stepped interface structures can be prepared by magnetron sputtering. Figure 1 illustrates the microstructure and phase structure of a typical Pd thin film with stepped interface structures (sample #4). SEM images of the film surface and cross-section are presented in Figure 1(a,a1). The sample shows a smooth surface, uniform grain pores, and polygonal grain morphology on the surface, with a statistically measured grain size of 92 ± 7 nm. The cross-section of the sample indicates that the film is composed of irregularly shaped columnar grains with a thickness of 628 ± 5 nm, which are tightly adhered to the substrate. Figure 1b presents a magnified image of the surface structure, clearly demonstrating that the surface of each particle has a stepped structure. Semi-annular stepped structures are observed on the surfaces of certain particles, as delineated by the red dashed lines in the figure, whereas annular stepped structures are present on others, as indicated by the yellow dashed lines.

Within the depth of field of SEM observation, the number of step layers on the particles ranges from three to six, with most being five layers. Statistical analysis indicates that the average spacing between the step layers on the particle surfaces is 13 ± 1 nm. The particles are separated by pores, which are uniformly distributed with a spacing of approximately 10 nm. Figure 1c displays the XRD pattern of the film sample. The diffraction peaks are located at 40.2°, 46.7°, and 68.2°, indicating the formation of a single-phase Pd with a face-centered cubic (FCC) structure.

TEM was employed to characterize the morphological features, lattice information, and cross-sectional grain structures with high clarity. Figure 1d shows the morphology of the sample cut by FIB, which has a length of approximately 10 μm. Figure 1e is the TEM image of the sample with the Si substrate at the bottom, the Pd thin film in the middle, and the Pt-C protective layer on top. The measured thickness of the film is 626 ± 3 nm, which is consistent with the result obtained from the SEM image. Figure 1(e1–e3) show the selected area electron diffraction (SAED) results of the three regions. The protective layer exhibits amorphous diffraction rings, indicating that it possesses a long-range disordered crystalline structure, which also ensures a clear distinction from the thin film sample. The thin film layer exhibits concentric ring patterns composed of spots, demonstrating its polycrystalline structure with crystallinity. By measuring the radii of the rings, it is confirmed that the sample has different crystal orientations with Pd [111], [200], [220], and [311]. The underlying Si substrate presents ordered lattice patterns, verifying its single-crystal structure with a crystal orientation of Si [011].

To clarify the atomic arrangement inside the film, high-resolution transmission electron microscopy (HRTEM) analysis was performed on the middle region of the sample, as shown in Figure 1f. Within the characterization area (60 nm × 80 nm), the sample exhibits complex lattice orientations. The regions marked with red dashed lines show three representative individual grains, with a size range of 10 nm to 15 nm. The interplanar spacing indicates that these are Pd grains with [111] and [200] orientations. In addition, the interplanar angles between adjacent grains vary in magnitude. The grains separated by blue dashed lines in Figure 1f exhibit a gradual change in orientation with small interplanar angles, while the grains in the bottom-right corner have larger interplanar angles.

**Figure 2:**
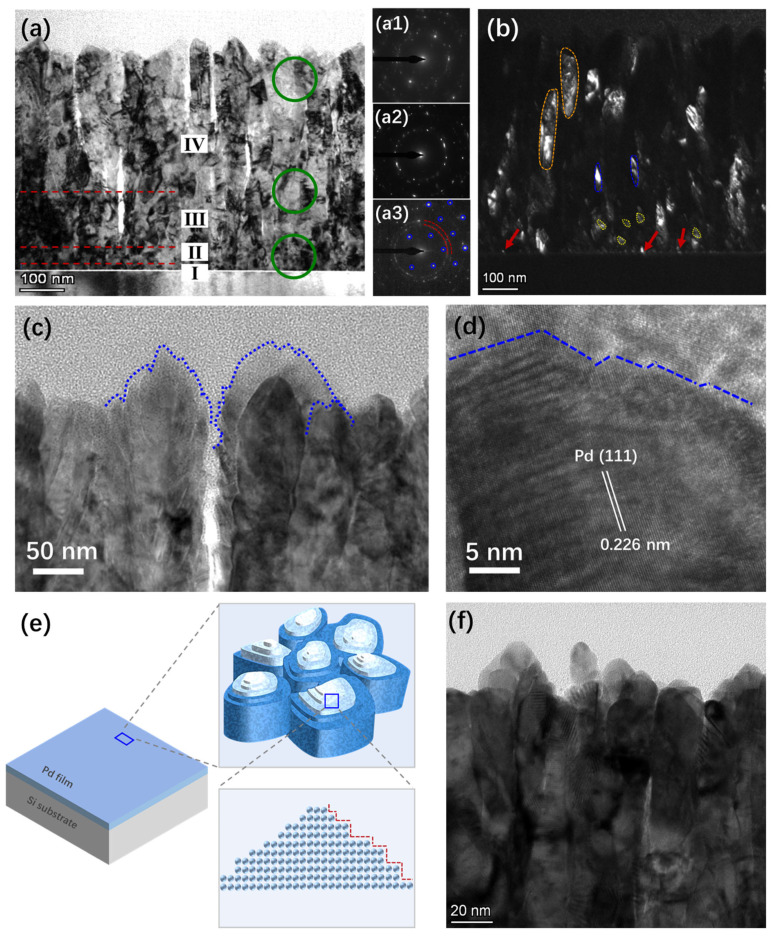
(**a**) TEM image of the Pd thin film sample, where regions I, II, III, and IV correspond to the local nanocrystalline region, equiaxed nanocrystalline region, non-equiaxed crystalline region, and columnar crystalline region, respectively. Insets (**a1**–**a3**) are SAED patterns of the green circled regions in the image. The blue areas in (**a3**) represent the Si diffraction lattice spots, while the red dashed rings correspond to the polycrystalline diffraction rings of Pd. (**b**) Dark-field TEM image. The red arrows indicate the axial crystal domain regions. The yellow, blue, and orange dashed lines indicate the equiaxed nanocrystalline, sub-columnar crystalline, and columnar crystalline regions, respectively. (**c**) TEM image and (**d**) high-resolution TEM image of the surface columnar crystals. The blue dashed lines illustrate the stepped structures on the sample interface. (**e**) Schematic diagram of the typical structure. (**f**) TEM image of the Pt film sample.

To investigate the vertical deposition process and atomic stacking behavior of the Pd thin film, more detailed TEM characterization was conducted. Figure 2a is the cross-sectional TEM image of the Pd thin film deposited on the Si substrate. The majority of grains near the upper surface of the sample exhibit a columnar structure, and the width of the columnar grains is dependent on their position. The portion near the surface is wider than that near the substrate. Thus, the columnar grains appear trapezoidal in shape in the cross-sectional view. In addition, some regions close to the Si substrate exhibit a unique microstructure. The SAED patterns acquired from the upper, middle, and lower regions along the axial direction of the film (as shown in Figure 2(a1–a3)) and the selected area dark-field TEM image (as shown in Figure 2b) clearly reveal the structural differences between the grains in the substrate region and those in the surface region.

In region I near the substrate, the dominant species are localized nanocrystals with ultra-small particle sizes, which are characterized by indistinct grain boundaries and random crystallographic orientations. In the dark-field image, axial crystal orientations present bright contrast, whereas off-axial crystal orientations exhibit dark contrast. Therefore, this region mostly appears as white dots, as indicated by the red arrows in Figure 2b. In region II, the structure is dominated by equiaxed nanocrystals (as indicated by the yellow dashed-line region in Figure 2b). SAED patterns of these two regions (as shown in Figure 2(a3)) reveal that they are mainly characterized by a multi-spot ring structure formed by nanocrystals. The widths of the local nanocrystalline region and the equiaxed crystalline region are approximately 10 nm and 65 nm, respectively. Above the equiaxed crystalline region (Region III), the grain orientations begin to exhibit anisotropy, as indicated by the blue region in Figure 2b.

The grains tend to align along the film growth direction (axial direction), while the radial dimensions show little variation. The weakened diffraction rings and reduced lattice spots in Figure 2a also confirm this observation, and the width of this region is approximately 150 nm. The anisotropy of the grains gradually increases with film growth. A higher axial atomic deposition rate than radial spreading rate yields a columnar crystal structure, as shown in the orange region of Figure 2b. Furthermore, the columnar grains near the film surface (Region IV) exhibit increasingly larger size and aspect ratio. The SAED pattern of this region (Figure 2(a1)) shows fewer lattice spots, higher intensity, and nearly vanished ring patterns. This phenomenon is attributed to the large grain size in this region, which results in fewer grains being enclosed by the selected area aperture and a more extensive distribution range of grains with identical orientations.

The interface structure of the film surface exerts a more direct impact on its hydrogen interaction performance. Consequently, we conducted a more detailed characterization of surface atomic stacking, the results of which are shown in Figure 2c,d. The structure is dominated by columnar crystals with vertical pores between adjacent grains. The upper surface of a single columnar crystal is generally conical and uneven, with step-like structures where the step heights range from 8 nm to 23 nm. High-resolution analysis of the step regions reveals that a large number of closely packed atomic steps are exposed on the serrated interface, as indicated by the blue dashed lines in Figure 2d.

Comprehensive multi-scale (top-view, cross-section, micro/nano) characterization yielded the structural information of the Pd thin films and the deposition process. Figure 2e presents a structural schematic diagram of the Pd thin film, showing that the sample possesses a cross-scale stepped interface structure. Despite its macroscopic flatness, the film exhibits a ring-shaped or semi-ring-shaped stepped surface morphology at the micro-nano scale. Furthermore, Pd atoms at the step edges demonstrate a distinct serrated stacking behavior at the lattice scale. To verify the universality of this technology, characterization was also performed on a Pt thin film, as shown in Figure 2f. Its microstructure is similar to that of the Pd thin film, and is thus not elaborated upon herein.

Typically, Pd thin films prepared via methods such as chemical synthesis or pulsed laser deposition exhibit a nearly spherical or snowman-like morphology [24,25]. In contrast, by regulating the magnetron sputtering parameters, we have obtained Pd thin films with a distinctive stepped microstructure. The formation mechanism of this stepped morphology is closely tied to the atomic deposition mode, as the overall growth process of the thin film is governed by the kinetic behavior of deposited atoms.

During the film deposition process, atoms on the surface of the Pd target are sputtered by high-energy Ar ions and subsequently deposited onto the substrate. Thin film formation from deposited target atoms involves nucleation, island growth and coalescence, and grain coarsening. Energetic sputtered atoms interact with substrate atoms and transform into weakly bonded adsorbed atoms via kinetic energy transfer [26]. These atoms then accumulate and migrate to form cluster-island structures. More sputtered atoms lead cluster-island structures to form local nanocrystals, which support epitaxy for small equiaxed nanocrystals at low-energy sites. High-energy deposition, recrystallization, and surface migration drive film thickening and columnar grain formation. Sputtered and deposited atom energy governs the film formation and structure. Both Pt and Pd possess FCC crystal structures with an ultralow lattice mismatch of only about 0.87%, and their intrinsic physicochemical properties (e.g., atomic radius, valence electron configuration) are well matched. Meanwhile, during magnetron sputtering deposition, they exhibit identical nucleation and growth modes, as well as consistent response tendencies to key process parameters, leading to convergent deposition kinetic behaviors. Given that Pd films display comparable microstructural evolution behavior to Pt films during deposition, while featuring lower cost and higher activity for typical electrocatalytic and hydrogen adsorption applications, the experimental results of Pd thin films are highlighted as the research focus in this work.

Stepped interface formation on the film surface arises from sputtered atom deposition and migration following columnar crystallization. As shown in Figure 2b, growth rates of individual crystal nuclei and island structures differ during the process of columnar crystal formation, causing competitive growth. Specifically, oriented nuclei achieve preferential growth via matching growth directions to incident atom directions and substrate lattice structure [19,27]. Enhanced atomic surface diffusion enables sufficient migration on columnar crystal tops and sides. Protruding regions preferentially capture incoming atoms and grow, forming a conical surface. According to crystal growth theory, sputtered atoms tend to diffuse along the surface to step or kink sites for adsorption and incorporation into primary crystals [28,29]. Atoms preferentially bind to existing surface steps, ultimately forming stepped zigzag structures on the crystal surface. Usually, these high-energy stepped planes grow faster and disappear preferentially in mature crystals [30]. Nevertheless, regulating deposition power and substrate temperature suppresses the surface diffusion energy of Pd atoms, preventing step atoms from acquiring enough kinetic energy for further growth toward a smooth surface and thus retaining the stepped interface.

### 3.2. Effects of Deposition Parameters on the Stepped Interface Structure

**Figure 3:**
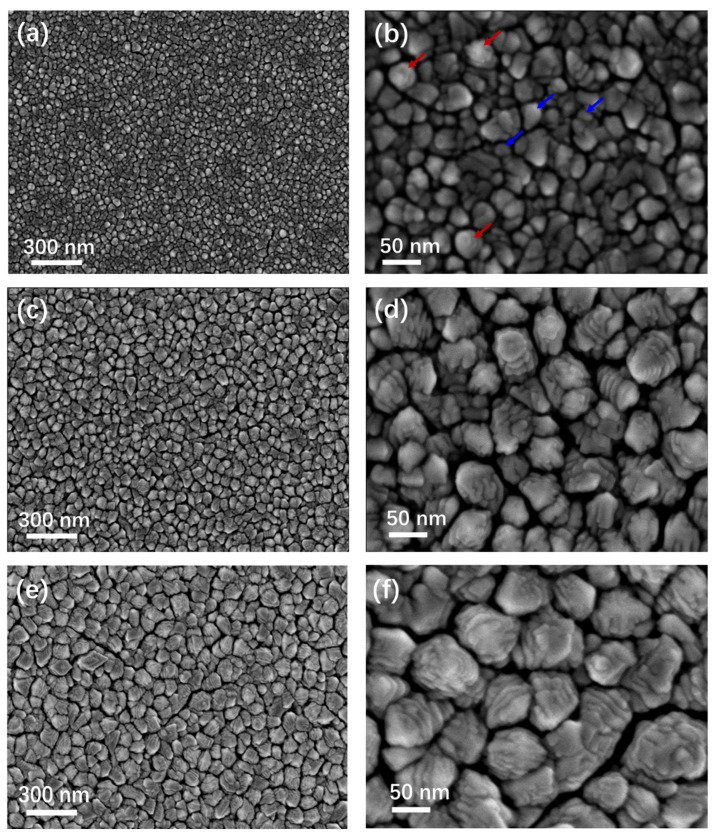
SEM images of Pd films deposited with different parameters. (**a**) Sample #1 at 50 W, (**c**) sample #2 at 100 W, and (**e**) sample #3 at 200 W. (**b**,**d**,**f**) are the high-magnification SEM images of (**a**,**c**,**e**). Blue and red arrows show particles without stepped interfaces and with ill-defined interfaces.

To clarify the correlation between deposition parameters (including sputtering power, substrate temperature, and argon pressure) and stepped interface structure, Pd thin films prepared under distinct deposition conditions were characterized and analyzed in this work. Figure 3 shows the SEM images of samples prepared under different deposition parameters. Figure 3a,c,e are the top-view SEM images of thin film samples (#1, #2, and #3, respectively) with the applied powers being 50 W, 100 W, and 200 W. Figure 3b,d,f are the corresponding magnified SEM images of the selected regions. As illustrated in Figure 3a, when the sputtering power was 50 W, the grain edges were rather smooth with an average grain size of 25 ± 2 nm. High-magnification observation indicates that most surface regions lacked a stepped interface structure (as shown by blue arrows), and only a few particles displayed a layered structure with blurred edges (as shown by red arrows). When the power increased to 80 W, the film surface exhibited a similar structure to that of this sample and is not further discussed. Figure 3c,d display the planar morphology of the 100 W sample, where almost all surface particles exhibited a stepped structure with some rough stepped planes. The statistical grain size was determined to be 60 ± 5 nm, and the step width was 11 ± 2 nm. At a sputtering power of 200 W, all particles on the film surface exhibited a stepped structure featuring smooth, predominantly ring-shaped steps. With the number of step layers ranging from 5 to 10, the grain size increased to 87 ± 7 nm. This suggests that sputtering power is a key factor that not only modulates the grain size of columnar crystals but also determines whether stepped structures can form and their density.

**Figure 4:**
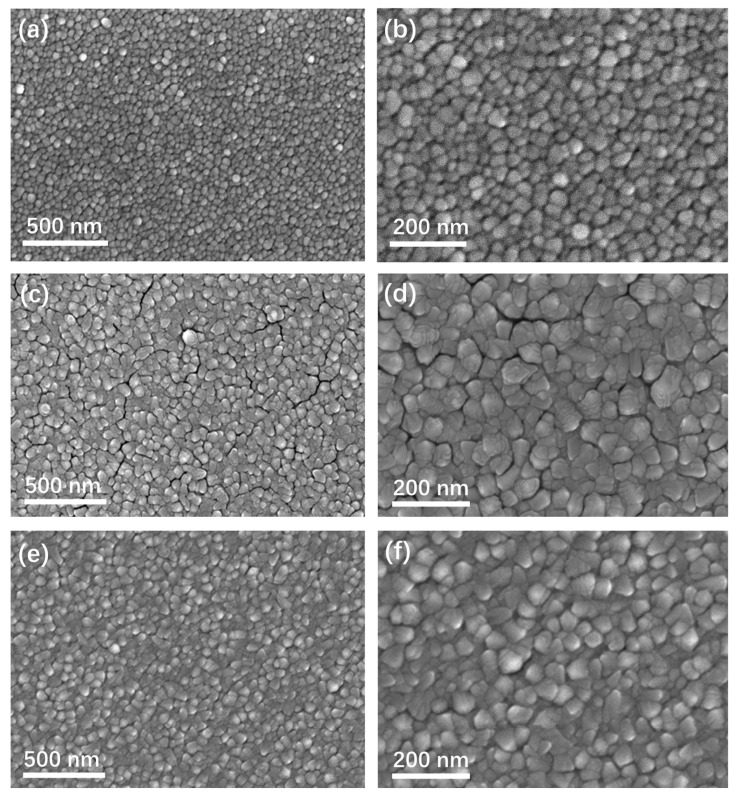
SEM images of Pd films deposited with different parameters. (**a**) Sample #5 at 50 W and 100 °C, (**c**) sample #6 at 200 W and 100 °C, (**e**) sample #7 at 100 W and 2 Pa. (**b**,**d**,**f**) are the high-magnification SEM images of (**a**,**c**,**e**).

In addition to the sputtering power, substrate temperature, and argon pressure are also key factors affecting the energy of sputtered and deposited atoms. Figure 4a,c are the SEM images of sample #5 and sample #6, sputtered at powers of 50 W and 200 W under high-temperature conditions. Figure 4e shows the SEM image of sample #7 deposited at an argon pressure of 2 Pa. Figure 4b,d,f are the corresponding magnified SEM images of the selected regions. As shown in Figure 4a, when the substrate temperature was 100 °C and the sputtering power was 50 W, the film surface was flat and free of pores, with the grain edges displaying smooth profiles. The statistical grain size was determined to be 39 ± 4 nm. High-magnification observation reveals a spherical surface morphology for this sample. In contrast to sample #1, elevating the substrate temperature at a 50 W sputtering power results in increased particle sizes and smoother grains, yet fails to generate stepped interface structures. Figure 4c demonstrates the structural changes caused by increased substrate temperature under 200 W sputtering power: the film surface consists of densely packed polygonal particles and displays a stepped structure with unclear step boundaries. The measured grain size of this sample is 89 ± 7 nm, showing a slight increase compared with sample #3. As illustrated in Figure 4e, decreasing the argon pressure from 3 Pa to 2 Pa resulted in an augmentation of grain size from 60 ± 5 nm to 63 ± 5 nm, accompanied by weakened surface stepped structures and higher film compactness.

**Figure 5:**
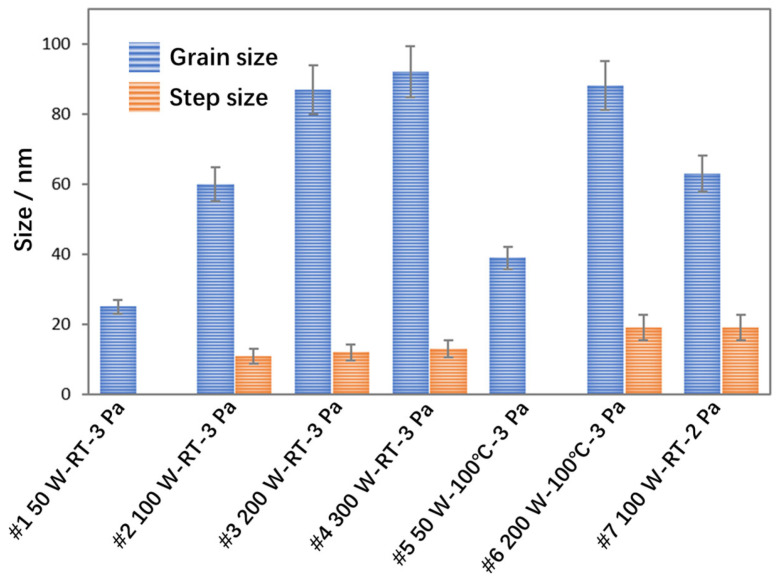
Statistical plot of grain size and step size of thin film samples under different deposition conditions.

To clearly contrast the effects of key parameters on the columnar grain size, as well as the step size and density, Figure 5 presents the data compilation and analysis. With increasing sputtering power, the grain size of Pd thin films rises, and more significant growth is observed at 100 W and 200 W. Specifically, the grain size at 100 W is 140% larger than that at 50 W, and the grain size at 200 W is 45% larger than that at 100 W, whereas the relative increase from 200 W to 300 W is merely 5.7%. This indicates that the sputtering power can modulate the columnar grain size, with the effect on grain size becoming weaker as the power increases. On the other hand, regarding the step size, no stepped structure was observed at 50 W, while stepped structures began to emerge at 100 W with a size of 11 nm, which is comparable to the pore size within the film. When the sputtering power was increased to 200 W, both the density and size of stepped interface structures increased, with a more pronounced rise in density. At 300 W, the stepped interface density decreased while the step size increased slightly from 11 nm to 13 nm. It can be concluded that the density and size of stepped interface structures can be tailored by controlling the sputtering power, with the power exerting a weaker effect on step size but a stronger influence on interface density.

Regarding the influence of substrate temperature, the most significant effect is upon grain size. Raising the substrate temperature results in an increase in grain size. As the temperature increases from room temperature to 100 °C, the grain size increases from 25 nm to 39 nm for the 50 W sample, and the grain size increases from 87 nm to 89 nm for the 200 W sample. The intense ion bombardment at high power counteracts the grain growth promotion effect of adatom diffusion induced by elevated temperature. Conversely, increasing substrate temperature does not generate stepped structures at low power, whereas at high power, it barely affects stepped interface morphology but reduces interface density. At a high sputtering power of 200 W, the sample #6 prepared at a substrate temperature of 100 °C exhibits an increased interlayer spacing accompanied by a reduced number of step layers compared with that fabricated at room temperature (sample #3). This is because elevated substrate temperature enhances the surface diffusion of deposited atoms, which fill the step gaps and thus reduce the number of step layers on the grain surfaces. A comparison of the experimental results of sample #2 and sample #7 shows that decreasing the argon pressure from 3 Pa to 2 Pa increased the film grain size from 60 nm to 63 nm and the step size from 12 nm to 19 nm. This indicates that reducing pressure leads to increases in both grain size and step size, with a more pronounced effect on step size.

During the thin film deposition process, the energy of the deposited adatoms dictates their migration ability and rate, which is determined by the substrate temperature and target power [31,32]. Adatoms are trapped at low-energy lattice sites, forming small localized nanocrystals. When these sites are saturated, remaining adatoms need higher energy to migrate to stable sites. Elevated substrate temperatures supply this energy, enabling adatoms to form continuous crystalline structures, thus improving crystalline quality. However, excessively high temperatures introduce excess energy that accelerates surface atom migration to stable positions, hindering zigzag atomic arrangements and reducing stepped interface density.

Target power energizes sputtered-substrate atom interactions. Higher sputtering power increases the kinetic energy and flux of sputtered atoms, thereby enhancing the surface diffusion of adatoms and promoting grain growth. Reducing both temperature and power thus reduces grain size, but low power inhibits surface atom migration, hindering zigzag stacking and eliminating stepped structures. Therefore, low-temperature and high-power deposition conditions are ideal for fabricating Pd thin films with small grains and abundant stepped interfaces.

During sputter deposition, sputtered atoms collide with Ar plasma prior to reaching the substrate, which constitutes a key mechanism underlying the dependence of film quality on Ar pressure. At low pressure, fewer gas molecules lead to less ionization and fewer ions bombarding the target, thus reducing the sputtering rate. Meanwhile, the longer mean free path of sputtered atoms enables them to acquire higher energy under the electric field before reaching the substrate, which is conducive to increasing the grain size, compactness, and step size of Pd films. In conclusion, sputtering power and substrate temperature dictate the energy of sputtered and deposited atoms, respectively. Moreover, argon pressure during sputtering directly modulates plasma density, thereby altering the collision paths and energy of sputtered atoms. Stepped microstructures in the thin films can be controllably tailored by tuning the critical parameters of magnetron sputtering.

### 3.3. Effects of Microstructure on Hydrogen Adsorption Properties

**Figure 6:**
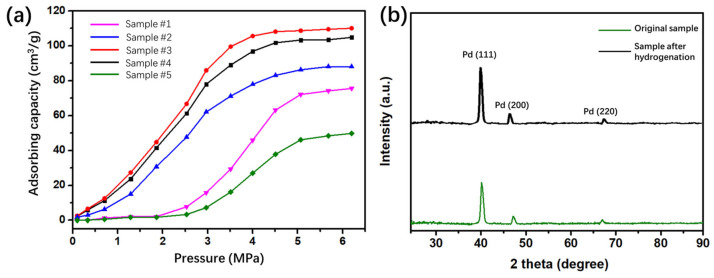
(**a**) Hydrogen adsorption capacity vs. pressure curves of samples #1 to #5 with different microstructures. (**b**) XRD patterns of sample #3 before and after hydrogen loading.

Based on the revealed microstructure of thin films with stepped interfaces and the key factors affecting the structure and size, we conducted hydrogen adsorption performance tests on Pd thin films with distinct characteristics, as well as phase composition characterization tests before and after hydrogen loading. The results are presented in Figure 6. Samples #1 to #5 with representative structures were chosen for hydrogen adsorption measurements, where Figure 6a shows the adsorption capacity-pressure curves of these samples. As demonstrated in the curves, the adsorption capacity of all samples increases with increasing test pressure, but their threshold pressures differ. Samples #1 and #5 have higher threshold pressures, with significant adsorption capacity growth occurring only above 2.5 MPa; whereas samples #2, #3, and #4 show a lower threshold pressure, with notable adsorption increases starting at pressures above 0.3 MPa. As test pressure increases, the adsorption capacity of different samples grows at distinct rates. The sample structure exerts a significant influence on the adsorption capacity. Sample #3 achieves the highest adsorption capacity of 110.06 cm^3^/g, which is 2.2 times higher than that of sample #5. The maximum adsorption capacity of sample #4 is 104.83 cm^3^/g, which is slightly lower than that of sample #3. Although Pd thin films have limited practical applications as hydrogen storage materials, they are indispensable for investigating the mechanisms underlying hydrogen-Pd interactions. Pd thin films with a stepped microstructure exhibit excellent hydrogen adsorption performance; specifically, the sample with the optimal step density and grain size achieves a hydrogen adsorption capacity of 0.983 wt.%. Compared with previous studies on multilayered materials where the Pd layer accounted for approximately 0.15–0.3 wt.% [33], the stepped microstructure improves the hydrogen adsorption capacity of samples by about 3.28 times. These results demonstrate that the atoms located at the stepped microstructures possess higher reactivity toward hydrogen.

To further reveal the mechanism by which stepped interface structures affect the hydrogen absorption, XRD experiments under the grazing incidence mode were conducted on sample #3 before and after the hydrogen loading reaction, as shown in Figure 6b. Distinct Pd (111), (200), and (220) peaks appear in the sample patterns before and after the reaction, which are characteristic of a polycrystalline FCC Pd phase. XRD results indicate that the thin films exhibit a [111] preferred orientation both before and after interaction with hydrogen. To further clarify the effect of hydrogen on the samples, a comparative analysis was conducted on the peak positions and full width at half maximum (FWHM) of the three main diffraction peaks. It was found that compared with the as-prepared samples, the peak positions of the sample after hydrogenation showed varying degrees of leftward shift. The Pd (111) and (200) peaks exhibit obvious left shifts from 40.13° to 39.72° and from 46.86° to 46.26°, respectively.

The diffraction peaks show a noticeable left shift after hydrogen loading, a direct consequence of lattice expansion induced by the formation of palladium hydride during hydrogen absorption by Pd. Upon intercalation into the octahedral and tetrahedral interstitial sites of the Pd lattice, hydrogen atoms exert repulsive forces on adjacent Pd atoms, which in turn induces isotropic lattice expansion. Based on Bragg’s equation, the increased interplanar spacing causes the peaks to shift toward lower 2θ angles, which is consistent with classical reports on Pd-H systems [34]. The Pd (111) and (200) planes bind to hydrogen more readily, resulting in a more significant swelling of their interplanar spacing. On the other hand, the FWHM of the Pd (111) peak for the pristine sample is 0.59°, while that of the hydrogen-loaded sample at the same peak position is 0.57°. The slight reduction in FWHM is attributed to high-temperature hydrogen loading. Ordinarily, inhomogeneous hydrogen distribution and differential strain across the grains and domains reduce the consistency of crystal plane orientation and weaken coherent XRD scattering. By contrast, elevated temperature enables uniform hydrogen diffusion, which mitigates inhomogeneous hydrogen distribution and differential strain [35]. Additionally, thermal effects repair intrinsic Pd lattice defects, including dislocations and vacancies, to reduce lattice distortion and improve lattice orderliness [36].

The hydrogen adsorption performance of Pd nanofilms is significantly affected by multiple factors, among which temperature, pressure, grain size, and surface microstructure play key roles. As the hydrogen pressure increases, the adsorption capacity of the sample rises progressively. This is due to the elevated collision frequency between hydrogen and the Pd surface at higher pressures, which allows more molecules to undergo adsorption and dissociation on the surface. Nevertheless, a saturation tendency appears when the pressure goes beyond a specific value. The reason lies in the finite active sites on the surface. After these sites are fully occupied, further pressure increments will exert little effect on the enhancement of loading capacity.

Particle size and microstructure also play an important role in hydrogen interactions. For the non-stepped-structure samples (#1 and #5), sample #1 exhibits a higher hydrogen loading capacity than sample #5, which can be attributed to its smaller grain size, rougher surface morphology, and intrinsic pore structure. In general, nano-Pd with a smaller grain size exhibits a larger specific surface area and more exposed surface atoms, which not only act as highly active adsorption sites for hydrogen atoms but also modulate the local electronic structure to lower the hydrogen adsorption energy barrier, thus synergistically enhancing hydrogen adsorption capacity and stability [37].

In addition, sample morphology, especially surface microstructure, significantly affects hydrogen loading capacity. The average grain sizes of samples #2, #3, and #4 are 60 nm, 87 nm, and 92 nm, respectively, which are larger than the 25 nm of sample #1. Nevertheless, samples #2, #3, and #4 exhibit superior hydrogen adsorption performance compared to sample #1. A key structural distinction is that samples #2, #3, and #4 possess stepped structures, while sample #1 does not. Thus, it can be reasonably inferred that the stepped structures increase the active sites for hydrogen interactions, offsetting the adverse effects of increased grain size. This phenomenon may be attributable to the unique interfacial crystalline structure of the sample. Specifically, atoms at the step sites have optimized electronic structures and higher energy, thus acting as active sites for hydrogen interactions. First-principles calculations have confirmed that such atomic-scale steps induce charge transfer effects and local density of states rearrangement, which modulate the Pd-H bond strength to enhance adsorption stability [38]. Surface stress release at steps introduces inhomogeneous strain fields, leading to distinct electronic structures and reactivity for terrace atoms with identical local coordination, and resulting in atomic site-specific enhancement of surface reaction activity [39].

Among the samples with stepped interface structures, sample #3 exhibits the highest hydrogen loading capacity, owing to its combination of small grain size and high-density stepped interface structures. For samples with stepped interfaces, interface density is one of the key factors influencing hydrogen interaction performance. It is characterized by the number of step layers on the surface of individual grains and the interlayer spacing. Sample #2 had an average interlayer spacing of approximately 11 nm, with most individual grains exhibiting 3–5 step layers; sample #3 had an average interlayer spacing of about 12 nm, where 5–10 step layers were predominant on individual grains; and sample #4 had an average interlayer spacing of roughly 13 nm, with 3–6 step layers on most individual grains. Considering the step layer number and interlayer spacing comprehensively, sample #3 exhibited the highest stepped interface density, followed by sample #4, and sample #2 exhibited the lowest. Accordingly, although samples #3 and #4 had slightly larger grain sizes than sample #2, their higher stepped interface density endowed them with superior hydrogen interaction performance.

These results confirm the positive effect of stepped interfaces on hydrogen loading capacity and demonstrate that regulating grain size and interface morphology through controlled preparation constitutes an effective strategy for enhancing hydrogen interaction performance. The undercoordinated atoms at stepped interfaces kinetically lower the activation energy barrier for H adsorption and dissociation [39]. Additionally, the distinctive local electronic structure and atomic configuration at step sites accelerate the charge transfer and mass transport of hydrogen species, thereby rendering these sites kinetically favorable active centers for hydrogen adsorption.

## 4. Conclusions

This work focuses on the deposition of Pd thin films and systematically investigates the relationship between stepped microstructure and hydrogen adsorption. The thin films with stable and wide-scale stepped interfaces were successfully prepared via atomic deposition under optimized magnetron sputtering parameters, showing excellent hydrogen interaction properties. A detailed analysis of the microstructures was conducted to clarify the growth mechanism of such thin films and the formation mechanism of stepped interfaces. Interactions occur between sputtered atoms and substrate atoms, accompanied by atomic migration and aggregation that lead to the formation of local nanocrystals on the substrate. As energy accumulates, equiaxed nanocrystal structures are formed. Columnar crystals are subsequently grown on these equiaxed nanocrystals via local epitaxy. The migration and diffusion of surface atoms on columnar crystals are moderately inhibited at optimal input energy, thereby yielding stable stepped interface structures. This study clarifies the relationship between the key parameters and the microstructural characteristics of Pd thin films, including grain size, morphology, as well as the density and size of stepped interfaces. Sputtering power, substrate temperature, and plasma density significantly affect the crystal structure size by regulating the movement and diffusion of sputtered atoms and adsorbed atoms. The Pd thin film deposited at room temperature with a power of 200 W exhibits the highest density of stepped interfaces and optimal hydrogen loading performance, with a hydrogen adsorption of 110.06 cm^3^/g, which is 2.2 times higher than that of conventional Pd thin films. The enhancement is attributed to its optimized electronic structure and surface energy, which effectively reduce the interaction threshold with hydrogen. Given the excellent hydrogen interaction performance of the tailored stepped Pd thin films, the findings pave the way for promising applications in hydrogen-related fields, such as high-sensitivity hydrogen sensors, lightweight electrochemical hydrogen storage devices, and selective separation layers in industrial hydrogen purification membranes. Furthermore, the stepped interfaces obtained in this study can be extended to other noble metal thin film systems, providing a new universal strategy for the development of high-performance hydrogen-interactive materials.

## Figures and Tables

**Table 1:** The deposition parameters for the film samples.

Sample Number	Deposition Parameters			
	Power (W)	Temperature (°C)	Ar Pressure (Pa)	Deposition Time (s)
#1	50	RT	3	2500
#2	100	RT	3	1500
#3	200	RT	3	600
#4	300	RT	3	300
#5	50	100	3	2500
#6	200	100	3	600
#7	100	RT	2	1500

## Data Availability

The original contributions presented in this study are included in the article. Further inquiries can be directed to the corresponding authors.
